# Vampiric Delusions in Treatment-Resistant Schizoaffective Disorder: A Case Report

**DOI:** 10.7759/cureus.115522

**Published:** 2026-08-31

**Authors:** Anthony H Nguyen, Patrick Vu, Kelly Chong, Caleb Mudd, Adam J Konen

**Affiliations:** 1 Psychiatry and Behavioral Sciences, University of Oklahoma Health Sciences Center, Oklahoma City, USA; 2 Psychiatry and Behavioral Sciences, University of Oklahoma Medical Center, Oklahoma City, USA

**Keywords:** auditory hallucinations, clinical vampirism, delusions, homelessness, long-acting injectable antipsychotics, methamphetamine associated psychosis, psychosis, renfield syndrome, schizoaffective disorder, violence risk

## Abstract

Clinical vampirism, or Renfield syndrome, is a rare psychiatric phenomenon characterized by the compulsion or desire to consume blood and has been described in association with psychotic and other psychiatric disorders. Reported presentations may include blood-drinking behaviors, urges to consume blood, and delusions or hallucinations involving blood.

This report details the case of a 28-year-old man with a history of treatment-resistant schizoaffective disorder and adolescent-onset polysubstance use who presented with prominent vampiric delusions. The patient endorsed urges to consume blood in the setting of persistent auditory hallucinations and religious delusions without acting upon these impulses. His presentation was complicated by psychosocial vulnerabilities, including chronic homelessness, medication nonadherence, and prior incarceration.

Following psychiatric evaluation and resumption of antipsychotic therapy, his homicidal ideation decreased, and he was transferred to an inpatient psychiatric facility for continued treatment.

This case highlights the relationship between clinical vampirism and underlying psychotic disorders and underscores the importance of addressing barriers to continuity of care when managing these rare presentations.

## Introduction

Clinical vampirism, referred to as Renfield syndrome, is an inconsistently defined and rare phenomenon in psychiatric literature, characterized by delusional beliefs, urges, or behaviors related to consuming blood [1]. Descriptions of vampirism have existed for centuries and have historically been associated with mythological and folkloric figures [2]. It is not recognized as a formal DSM-5-TR diagnosis, with fewer than 100 cases described in psychiatric literature [1]. Patient presentations vary widely, ranging from intrusive urges or delusions of self-directed blood consumption to consuming the blood of others [1]. These presentations have often been described within the context of psychotic disorders, personality disorders, and paraphilic disorders [1]. The heterogeneity of patient presentations and scarcity of published cases have led to ongoing debate regarding whether clinical vampirism is a distinct diagnosis or a symptom of underlying psychiatric pathology [1]. 

This report presents a case of a young adult male patient with a history of treatment-resistant schizoaffective disorder despite multiple antipsychotic treatment trials and comorbid substance use who developed clinical vampirism as a manifestation of chronic psychosis. This case contributes to the existing literature examining clinical vampirism as a feature of psychosis and highlights the importance of contextualizing blood-drinking behaviors within established psychiatric diagnoses rather than as an isolated syndrome. 

## Case presentation

A 28-year-old male patient with a history of treatment-resistant schizoaffective disorder and adolescent-onset polysubstance use was transferred alone from an outside emergency department to the neurological intensive care unit for evaluation of a suspected left cerebellar lesion after reporting transient, unspecified abnormal eye movements and concern for elevated blood pressure prior to presentation. The etiology of these symptoms was undocumented. A noncontrast computed tomography scan obtained at an outside hospital demonstrated a left cerebellar hyperdensity concerning for an acute hemorrhage. Upon arrival, he was awake, cooperative, and alert, and oriented to person, place, and time. Blood pressure and vital signs were within normal limits and remained stable during the hospital course. Physical examination was unremarkable, and neurologic exam did not demonstrate focal deficits, nystagmus, or other abnormal eye movements. Computed tomography imaging of the brain revealed a left cerebellar hyperdensity. However, inpatient documentation noted that the lesion represented a hereditary cavernous hemangioma, first documented in a magnetic resonance imaging study from 2019, with no hemangioma-directed treatment documented at the time of presentation. No other significant radiologic findings were documented during admission. Laboratory investigations (Table 1), including a basic metabolic panel, complete blood count, urinalysis, and urine toxicology screening, were unremarkable. 

**Table 1:** Vital signs and laboratory findings CBC: complete blood count, BMP: basic metabolic panel, UA: urinalysis, UDS: urine drug screen, PCR: polymerase chain reaction, ECG: electrocardiogram.

Measure	Result
Heart rate	84 bpm
Respiratory rate	20 breaths/min
Blood pressure	123/85 mmHg
Body mass index	22.37 kg/m²
Height	1.829 m
CBC	Within normal limits
BMP	Within normal limits
Fingerstick blood glucose	129 mg/dL
UA	Normal
UDS	Negative
SARS-CoV-2 PCR	Negative
Influenza A/B PCR	Negative
ECG	Normal sinus rhythm; QTc = 386 ms

During hospitalization, he expressed homicidal ideation with an urge to “bite people’s arteries to drink their blood.” He reported having these urges for the past 10 years; however, the frequency of these urges was not documented. He claimed that a “demon” tells him to act on these thoughts and that “Jesus spoke about feeding [his] flesh.” Consult-liaison psychiatry was consulted, and a mental status exam was conducted. He was observed to have an anxious mood with flat affect, poor insight and judgement, active auditory hallucinations, and religious thought content. No additional auditory hallucinations were documented. Other delusions included a belief that the FBI was after him because he killed a funeral director and hid his body.

His medical history is significant for HIV infection at age 19, managed with bictegravir/emtricitabine/tenofovir alafenamide 50/200/25 mg daily. At presentation, his HIV was well controlled, with an undetectable viral load <20 copies/mL and a CD4 count of 883 cells/µL. No HIV-related complications or opportunistic infections were documented.

Collateral history from the patient's mother and chart review revealed a reported diagnosis of schizoaffective bipolar-type disorder with his first psychotic episode at the age of 17 and an 11-year history of methamphetamine, marijuana, and benzodiazepine use (Table 2). When not using substances, he expressed thoughts of harming others and drinking their blood because a “demon told him to.” He reported more than 20 past psychiatric hospitalizations, a previous suicide attempt at age 19, and worsening psychosis within the past year. The circumstances and timing of past psychiatric hospitalizations and circumstances surrounding the suicide attempt were not documented. He is currently homeless and has been arrested at least three times for offenses including public intoxication, assaultive behavior toward law enforcement, and attempted theft. He does not see a psychiatrist regularly. He has undergone extensive psychotropic medication trials (Table 3). At an unspecified time prior to admission, a restraining order was issued following multiple unspecified threats toward the patient’s mother and her husband, limiting the availability of collateral history from his mother. Family history is notable for depression, substance use, seizures, and stroke.

**Table 2:** Psychosocial history BID: two times a day, TID: three times a day.

Category	Information
Psychiatric diagnosis	Schizoaffective disorder, bipolar type
Current psychiatric treatment	Inconsistent psychiatric follow-up
Current psychotropic medications	Risperidone 2 mg BID; Quetiapine 200 mg TID
Psychiatric hospitalizations	More than 20
Substance use history	Methamphetamine, marijuana, benzodiazepines
Childhood medical history	None reported
Living situation	Homeless
Highest education attained	11th grade
Employment	Unemployed

**Table 3:** Past psychotropic medications Dosages and frequencies were provided if found on chart review.

Medications	Dosage	Frequency	Outcomes
Aripiprazole	Unknown	Unknown	Discontinued due to oversedation
Quetiapine	200 mg	Three times daily	Home medication
Ziprasidone	Unknown	Unknown	Discontinued for unknown reasons
Lurasidone	Unknown	Unknown	Discontinued due to persistent auditory hallucinations
Asenapine	Unknown	Unknown	Discontinued due to persistent auditory hallucinations
Chlorpromazine	Unknown	Unknown	Discontinued for unknown reasons
Lithium	300 mg	Unknown	Discontinued for unknown reasons
Valproate	500 mg	Twice daily	Home medication
Olanzapine	15 mg	Twice nightly	Well tolerated but discontinued for unknown reasons
Risperidone	2 mg	Twice daily	Home medication
Paliperidone palmitate	234 mg/1.5 mL IM	Monthly	Discontinued due to worsening agitation and homicidal ideation

Following psychiatric evaluation, the differential diagnoses included primary psychotic disorder given his established history of schizoaffective disorder and substance-induced psychosis given his history of polysubstance use.

Given the acute psychotic presentation, management focused on pharmacologic stabilization, and psychotherapy was not initiated. Pharmacological treatment included resuming psychiatric medications, including risperidone 2 mg twice daily, valproate 500 mg twice daily, and quetiapine 200 mg three times daily. For home medications, bictegravir/emtricitabine/tenofovir alafenamide 50/200/25 mg daily was resumed. Quetiapine was discontinued the next day due to lack of efficacy. Oral lorazepam 2 mg and oral haloperidol 5 mg were used in combination as needed for agitation. By day 3, the patient was calm without observed agitation and denied current homicidal ideation, although he continued to endorse auditory hallucinations. The patient was transferred that day to an inpatient psychiatric facility on risperidone 2 mg twice daily, fluoxetine 20 mg daily, and divalproex 500 mg daily without acute medical concerns at transfer. The patient was lost to follow-up after discharge, and no subsequent clinical information was available (Table 4).

**Table 4:** Case timeline

Time	Clinical events
Age 17	First reported psychotic episode; subsequently diagnosed with schizoaffective disorder, bipolar type
~10 years before current admission	Onset of recurrent urges to harm others and drink their blood, reportedly associated with auditory hallucinations involving a “demon”
Prior to current admission	Extensive psychiatric history including approximately 20 psychiatric hospitalizations, a suicide attempt at age 19, polysubstance use, housing instability, and inconsistent psychiatric follow-up
Day 0	Presented after reported transient abnormal eye movements and concern for reported elevated blood pressure. Noncontrast CT demonstrated a left cerebellar hyperdensity concerning for acute hemorrhage, prompting transfer to the neurological ICU
Day 1: neurologic/medical evaluation	The patient was alert and oriented with stable vital signs and no focal neurologic deficits, nystagmus, or abnormal eye movements. Repeat neuroimaging demonstrated the known left cerebellar lesion, previously documented as a cavernous hemangioma. CBC, BMP, urinalysis, and urine toxicology were unremarkable. No acute neurologic process was identified.
Day 1: psychiatric evaluation	The patient expressed homicidal ideation and urges to bite others’ arteries and drink their blood. He reported auditory hallucinations with religious content and other persecutory/delusional beliefs. Consult-liaison psychiatry evaluated the patient
Day 1-2: treatment	Home risperidone 2 mg twice daily, valproate 500 mg twice daily, and quetiapine 200 mg three times daily were restarted. Quetiapine was discontinued the following day because of lack of efficacy. Lorazepam 2 mg and haloperidol 5 mg were administered as needed for agitation
Day 3: disposition	The patient was calm without observed agitation and denied homicidal ideation but continued to endorse auditory hallucinations. The patient remained medically stable and was transferred to an inpatient psychiatric facility on risperidone 2 mg twice daily, fluoxetine 20 mg daily, and divalproex 500 mg daily
Follow-up	The patient was lost to follow-up after discharge; subsequent clinical status, treatment, and quality of life were unavailable

## Discussion

Prior case reports have described vampiric ideation in the context of schizophrenia-spectrum disorders, often in association with delusions or command hallucinations. One case report described auto-vampirism in schizophrenia in association with purification rituals and command auditory hallucinations [2]. Another case report reported vampiric delusions in treatment-resistant schizophrenia, with command hallucinations instructing the patient to attack others to consume their blood [3]. These cases parallel this patient’s presentation, in which vampirism was a manifestation of a broader psychotic syndrome.

The patient’s initially reported eye movements and hypertension raised concern for an acute neurologic process, particularly in the setting of a suspicious cerebellar lesion on neuroimaging. However, the eye movements were not reproduced on subsequent neurologic examination, and the reported hypertension was not documented on serial vital-sign measurements, which remained within normal limits. A negative urine drug screen provided no evidence of recent substance exposure, although his history of chronic polysubstance use warranted consideration of substance-related contribution to his psychosis. The presence of active command hallucinations, flat affect, and an established schizophrenia-spectrum disorder favored primary psychotic disorder as the principal explanation for his presentation. An isolated delusional disorder was less consistent with this broader psychotic presentation.

According to the Diagnostic and Statistical Manual of Mental Disorders, Fifth Edition, Text Revision (DSM-5-TR), the criteria for paraphilic disorders require recurrent patterns of atypical sexual arousal that cause significant distress, impairment, harm, or risk of harm [4]. Although Renfield syndrome has been loosely associated with paraphilia, no sexual arousal associated with the patient's urges was documented. Although vampirism has historical associations with mythology and folklore, no culturally sanctioned explanation for the patient’s beliefs or urges was documented. Instead, his urges occurred alongside command auditory hallucinations and delusional thought content, supporting their interpretation within a psychotic disorder rather than a paraphilia disorder or culturally shared phenomenon.

This patient’s history of substance abuse contrasts with previous reports of vampirism. In this case, the patient’s long-term use of methamphetamine is an additional risk factor that may have contributed to his chronic delusions and psychosis. Methamphetamine-associated psychosis is thought to involve dopaminergic dysregulation via inhibition of DAT and VMAT2 pathways [5]. The clinical presentation of methamphetamine-associated psychosis overlaps with schizophrenia-spectrum disorders, with the most frequently reported symptoms being persecutory delusions and auditory hallucinations [6]. However, only a small proportion of methamphetamine users experience persistent psychosis despite abstinence [7]. Persistent psychosis has been proposed to reflect an interaction between underlying susceptibility to psychiatric illness and methamphetamine-associated neurotoxicity [6].

Patients with psychosis have an increased risk of violence, particularly among those with a history of criminal behavior, substance use, or suicide attempts [8,9]. This patient exhibited all three risk domains. In addition, homeless individuals with mental illness are at higher risk of incarceration and face significant barriers to healthcare access [8,10,11].

Like previous reports of Renfield syndrome, the patient had treatment-resistant schizoaffective disorder with extensive trials of antipsychotics [2,3,12]. Evidence regarding the treatment of Renfield syndrome remains limited, with management involving antipsychotics for underlying psychosis in previous case reports [2,3,12]. Clozapine, an atypical antipsychotic, has demonstrated efficacy against treatment-resistant schizophrenia and has been reported to reduce vampiric delusions in an individual case [3,12]. However, it requires close medical follow-up due to the risk of neutropenia [3]. During hospitalization, his home risperidone, valproate, and quetiapine were resumed for mood stabilization and psychosis. In patients with unstable living conditions and schizophrenia, long-acting injectable (LAI) antipsychotics may improve medication adherence and decrease relapse [13]. The patient had been previously treated with paliperidone palmitate, which was discontinued due to worsening agitation and homicidal ideation. The rationale for not initiating an alternative LAI during the present hospitalization was not documented. The potential adherence benefits of LAIs are an important consideration for patients who face barriers to receiving consistent psychiatric care, incarceration, and housing instability. Further research is needed to assess treatment responses in patients with vampiric delusions and determine which psychosocial and pharmacological interventions improve long-term outcomes.

## Conclusions

This case supports the interpretation of Renfield syndrome as a rare manifestation of an underlying psychotic disorder rather than a discrete diagnostic entity. In this patient, the presence of active auditory hallucinations, religious delusions, and homicidal ideation within an established schizoaffective disorder favored a primary psychotic etiology over an isolated delusional or paraphilia disorder. Although his history of polysubstance use warranted consideration of a substance-induced etiology to his psychosis, the broader clinical presentation supported an underlying primary psychotic disorder. His history of homelessness, incarceration, substance use, and inconsistent medication adherence and psychiatric care complicated the management of his chronic psychosis. Given the limited evidence on the treatment of clinical vampirism, management should focus on the underlying psychiatric disorder while addressing barriers to continuity of care and medication adherence. Further research is required to characterize these presentations and their response to pharmacologic and psychosocial interventions. 
